# Stomatal Responses to Light, CO_2_, and Mesophyll Tissue in *Vicia faba* and *Kalanchoë fedtschenkoi*

**DOI:** 10.3389/fpls.2021.740534

**Published:** 2021-10-27

**Authors:** Mauro G. Santos, Phillip A. Davey, Tanja A. Hofmann, Anne Borland, James Hartwell, Tracy Lawson

**Affiliations:** ^1^School of Life Sciences, University of Essex, Wivenhoe Park, Colchester, United Kingdom; ^2^OSFC, Scrivener Dr, Pinewood, Ipswich, United Kingdom; ^3^School of Natural and Environmental Sciences, Devonshire Building, Newcastle University, Newcastle upon Tyne, United Kingdom; ^4^Department of Biochemistry and Systems Biology, Institute of Systems, Molecular and Integrative Biology, University of Liverpool, Liverpool, United Kingdom

**Keywords:** stomata, CAM, mesophyll, *Vicia faba*, *Kalanchoë fedtschenkoi*, conductance, stomatal dynamics

## Abstract

The responses of stomatal aperture to light intensity and CO_2_ concentration were studied in both *Vicia faba* (C_3_) and *Kalanchoë fedtschenkoi* (Crassulacean acid metabolism; CAM), in material sampled from both light and dark periods. Direct comparison was made between intact leaf segments, epidermises grafted onto exposed mesophyll, and isolated epidermal peels, including transplantations between species and between diel periods. We reported the stomatal opening in response to darkness in isolated CAM peels from the light period, but not from the dark. Furthermore, we showed that C_3_ mesophyll has stimulated CAM stomata in transplanted peels to behave as C_3_ in response to light and CO_2_. By using peels and mesophyll from plants sampled in the dark and the light period, we provided clear evidence that CAM stomata behaved differently from C_3_. This might be linked to stored metabolites/ions and signalling pathway components within the guard cells, and/or a mesophyll-derived signal. Overall, our results provided evidence for both the involvement of guard cell metabolism *and* mesophyll signals in stomatal responses in both C_3_ and CAM species.

## Introduction

The waxy leaf surface is almost impermeable to carbon dioxide and water and therefore nearly all gaseous exchange between the leaf interior and the external environment passes through the stomatal pores on the leaf surface (Lawson, 2009). Stomata open and close in response to changes in both external environmental and internal plant signals (e.g., Mott, 1988; Outlaw, 2003; Vavasseur and Raghavendra, 2005; Shimazaki et al., 2007; Lawson, 2009). The nuanced control of stomatal aperture ensures sufficient carbon dioxide (CO_2_) uptake for photosynthesis, as well as maintaining an appropriate water (H_2_O) status and leaf temperature (Lawson and Blatt, 2014). In species with C_3_ and C_4_ photosynthetic metabolism, stomata open in response to low CO_2_ concentration, high light, and low VPD, whereas closure is driven by the reverse, high CO_2_ concentration, low light, and high VPD (Outlaw, 2003; Vavasseur and Raghavendra, 2005; Shimazaki et al., 2007; Lawson, 2009).

It is well-established that stomatal conductance (*g*_*s*_) correlates with mesophyll rates of photosynthesis under a range of different conditions (Farquhar and Wong, 1984; Wong et al., 1979; Mansfield et al., 1990; Buckley et al., 2003). This helps to maintain an appropriate balance of CO_2_ uptake with water loss (often referred to as instantaneous water use efficiency). Until recently it was thought that [CO_2_] concentration in the sub-stomatal cavity, internal [CO_2_] (*C*_*i*_) co-ordinated stomatal behaviour with mesophyll demands for CO_2_. For example, when irradiance increases CO_2_ consumption by the mesophyll, stomata will respond to the decrease in *C*_*i*_ by opening (Mott, 1988). Conversely, when photosynthesis is reduced due to a changing environmental factor, the higher *C*_*i*_ results in stomatal closure. However, several studies have suggested that stomatal responses to changing *C*_*i*_ are too small to account for the differences in observed *g*_*s*_ in response to light (Raschke, 1975; Farquhar et al., 1978; Sharkey and Raschke, 1981; Farquhar and Sharkey, 1982; Morison and Jarvis, 1983; Ramos and Hall, 1983; Mott, 1988). These findings had led to the proposal that there must be an alternative signal (see Lawson et al., 2018).

Stomata in Crassulacean acid metabolism (CAM) plants operate differently. They open at night when there is no light, and this facilitates CO_2_ uptake when evaporative demand is low. In addition, they close during the day when light intensity and ambient temperatures are high, thereby minimising water loss through transpiration and optimising water use efficiency (Males and Griffiths, 2017). During the night in a CAM photosynthetic tissue, phosphoenolpyruvate carboxylase (PEPC or PPC) in the mesophyll draws down CO_2_, as it functions as the primary nocturnal carboxylase for atmospheric CO_2_ fixation (Borland et al., 2009). This nocturnal draw-down is hypothesised to drive stomatal opening in the dark. Atmospheric and respiratory CO_2_ fixed at night by PPC is stored as malic acid in the vacuole, reaching a maximum concentration at dawn (Borland et al., 2009). During the light period, the stored malate is transported out of the vacuole and decarboxylated, and the CO_2_ released increases *C*_*i*_ (Males and Griffiths, 2017). This increase in the light period in *C*_*i*_ due to malate decarboxylation has been proposed to drive stomatal closure in the light (Cockburn et al., 1979; Spalding et al., 1979; Borland et al., 1998; von Caemmerer and Griffiths, 2009). In general, as in C_3_ plants, stomatal responses in CAM plants have been attributed to changes in *C*_*i*_ (von Caemmerer and Griffiths, 2009; Males and Griffiths, 2017).

In addition, it is increasingly clear that the circadian clock is likely to play an important role in CAM stomatal regulation (Boxall et al., 2005, 2020; Hubbard and Webb, 2015). In a seminal paper, von Caemmerer and Griffiths (2009) manipulated external CO_2_ concentration and demonstrated stomatal closure in CAM-performing *Kalanchoë daigremontiana* leaves in the light period. The fact that the stomata closed in the light despite low *C*_*i*_ suggested that *C*_*i*_ was not the sole factor driving CAM stomatal responses and that another signal, possibly from the endogenous circadian clock, interacted with *C*_*i*_ to influence stomatal behaviour (von Caemmerer and Griffiths, 2009).

There is evidence that metabolites also play an important role in stomatal regulation. For example, when nocturnal CO_2_ fixation and associated malic acid synthesis and accumulation were reduced by restricting CO_2_ supply to *K. daigremontiana* leaves in the dark period, it was discovered that the adjusted metabolic status of the leaf could override the dawn-phased, circadian clock-controlled disappearance of the regulatory protein kinase responsible for making PPC less sensitive to feedback inhibition by malate, namely PPC kinase (PPCK) (Borland et al., 1999). In addition, transgenic gene silencing approaches have been used to generate CAM loss-of-function mutants in the CAM model species *Kalanchoë fedtschenkoi* and *Kalanchoë laxiflora* (e.g., Hartwell et al., 2016). Physiological, metabolic, and molecular phenotypic responses have been characterised for *Kalanchoë* mutants lacking the primary carboxylase PPC1, its circadian clock-controlled, nocturnal regulator PPCK1, plastidic α-glucan phosphorylase (PHS1) required for starch breakdown for PEP provision in the dark, and two key steps in the malate decarboxylation pathway that operates in the light period (Dever et al., 2015; Boxall et al., 2017, 2020; Ceusters et al., 2021). These mutants displayed either no dark atmospheric CO_2_ fixation, or reduced nocturnal CO_2_ fixation, but they still displayed decreased stomatal conductance in the middle of the light period (e.g., Boxall et al., 2020). The CAM mutants also revealed likely cross-talk between CAM-associated metabolites and both the leaf circadian clock and the light/dark regulation of guard cell genes known to be involved in stomatal opening and closing (Boxall et al., 2020).

Further evidence that questioned the role of *C*_*i*_ in stomatal behaviour in C_3_ plants came from experiments in which stomata responded to increasing light even when *C*_*i*_ was held constant (Messinger et al., 2006; Lawson et al., 2008).

Together, these findings led to the hypothesis that a diffusible mesophyll signal co-ordinates stomatal behaviour with mesophyll demands for CO_2_ (Lee and Bowling, 1992, 1995; Mott et al., 2008; Mott, 2009). The idea for a mesophyll signal was initially proposed by Heath and Russell in 1954, who postulated that stomatal behaviour was influenced by an indirect chemical or electrical signal transmitted from mesophyll or epidermal cells. Further support for a diffusible signal from the mesophyll was provided by the study of Lee and Bowling (1993), who demonstrated a stomatal response when isolated peels were incubated in the presence of mesophyll cells or chloroplasts from an illuminated leaf, but not when mesophyll tissue was not present, or when chloroplasts were used from dark-adapted material (see Lawson et al., 2018). Later studies suggested a photosynthetic intermediate or metabolite (Wong et al., 1979; Grantz and Schwartz, 1988; Lee and Bowling, 1992), specifically one that balances photosynthesis between Rubisco and electron transport limitation (Wong et al., 1979; Messinger et al., 2006). Support for the role of an active mesophyll-driven signal in stomatal responses has been provided from experiments carried out on epidermal peels in which the influence of the mesophyll had been removed. These studies have demonstrated either no effect or a slower response, of stomata to red light and/or [CO_2_] (Lee and Bowling, 1992; Olsen and Junttila, 2002; Roelfsema et al., 2002), as compared with responses reported in intact leaves (Mott et al., 2008).

However, as pointed out by the study of Fujita et al. (2013), utilising isolated epidermises floated on buffer solutions makes it difficult to track the same stoma due to movement in the buffer and the buffer permeating into sub-stomatal cavities, which are normally in contact with air. To overcome this, the study of Fujita et al. (2013) used a solid gellan gum matrix incorporating buffers, which was believed to mimic a leaf structure more closely. These experiments showed that buffer-filled cavities affected stomatal responses due to a lack of gaseous diffusion. To overcome the problems associated with using peels floated on the buffer, the study of Mott et al. (2008) used a unique epidermis–mesophyll transplantation experimental approach. The epidermis from one leaf was peeled and placed on the mesophyll belonging to either the same species or another species. Stomatal responses to changes in irradiance and [CO_2_] were different when the epidermises were assayed in isolation as compared to those in contact with mesophyll tissue (Mott et al., 2008). By injecting various solutions into the leaf, the work of Sibbernsen and Mott (2010) suggested that the mesophyll signal must be gaseous, and, following later experiments, proposed vapour phase ions as the entities responsible for mesophyll control of guard cells and stomatal aperture (Mott and Peak, 2013; Mott et al., 2014). The study of Fujita et al. (2013) further tested this hypothesis by using different combinations of cellophane and polyethylene films inserted between an epidermal peel and the gel-based support medium. Only aqueous solutes could pass through the cellophane, whereas only gases could pass through the polyethylene film. No stomatal response to CO_2_ was observed when using polyethylene films. However, a response was reported when using cellophane film, which led the authors to conclude that the mesophyll to guard cell signal must be aqueous (Fujita et al., 2013).

Several studies have examined stomatal behaviour in epidermises from one leaf placed onto mesophyll from a different leaf or species (Mott et al., 2008; Shope et al., 2008; McAdam and Brodribb, 2012; Fujita et al., 2013). The study of McAdam and Brodribb (2012) used a grafting approach (called xenografts) to assess differential influences of mesophyll on seed plants relative to ferns and showed that stomatal closing in response to light was impaired in isolated peels of seed plants but not the older ferns or lycophytes (McAdam and Brodribb, 2012). However, to date, no study has investigated the stomatal responses of an epidermis transplanted onto mesophyll of a species with different photosynthetic metabolism. Specifically, we examined stomatal responses to changes in irradiance and [CO_2_] in the epidermis of the C_3_ plant *Vicia faba* when placed on the mesophyll of the CAM species *K. fedtschenkoi* and *vice versa*. This unique approach has several distinct advantages. Each photosynthetic type exhibits an opposing stomatal response to the light and dark periods, and the photosynthetic mesophyll cells of C_3_ and CAM species have markedly different metabolite pools at different times in the light and dark. Thus, we established an experimental system that provided novel insights into the question of whether or not a mesophyll-derived signal influences stomatal aperture responses. For example, the question “Will C_3_ epidermal stomata sampled in the light still open in response to light when transplanted onto CAM mesophyll from the light?” was investigated in this study.

## Materials and Methods

### Plant Material and Growth Conditions

*Vicia faba* (L.) (C_3_) seed and *Kalanchoë fedtschenkoi* (Hamet et Perrier) (CAM) clonal stem cuttings were grown in two identical controlled environments (PG660, Sanyo, UK) using 24 h cycles of 12-h light [390 (±10) μmol m^−2^ s^−1^ at the top of the canopy], 25°C, and 12-h dark, 18°C, and a constant vapour pressure deficit of 1(±0.1) kPa in the light and dark. In the first controlled environment, the 12-h light period was from 8:00 a.m. to 8:00 p.m., which will be known as the “light” chamber. The second controlled environment chamber had an inverted light and dark cycle, such that the 12-h light period was from 8:00 p.m. to 8:00 a.m., and thus, the chamber was in the 12-h dark period when the “light chamber” was in its 12-h light period from 8:00 a.m. to 8:00 p.m. This second chamber is hereafter referred to as the “dark” growth chamber. Plants were grown in 0.5 L pots containing peat-based compost (Levington F2+S, ICL, UK) and were watered daily. *V. faba* plants used during the experimental period were at least 4-weeks post-emergence, and *K. fedtschenkoi* were at least 2 months old and had acclimated to their respective controlled environment for at least 2 months. The youngest fully expanded leaves were used from the *V. faba*, whilst mature leaves (older than 6 leaf pairs down from the apical meristem) were used from *K. fedtschenkoi* to ensure the leaves were CAM, as younger leaves have been shown to operate as C_3_, with a gradual developmental progression to full CAM in leaf pair six and older (Jones, 1975; Hartwell et al., 1999; Borland et al., 2009; Boxall et al., 2020).

### Preparation of Leaf Material

Leaf segments at 11 × 15 mm in size were cut from the central mid lamina of selected leaves and exposed mesophyll was generated by peeling away the abaxial epidermis with the aid of broad-tip tweezers. Isolated epidermises were prepared in the same way from the lower surface of leaves of both species and were washed with distilled water after peeling. Visual examination of these epidermises showed that essentially no mesophyll cells remained. The prepared material was immediately placed on a 3 cm diameter philtre paper saturated with distilled water, or if used as an isolated epidermis, placed on philtre paper saturated with incubation media following the methods of Mott et al., 2008 (3 ml of 50 mM KCl and 1 mM CaCl_2_). It should be noted that no buffers were used to prevent counteraction of the membrane H^+^-ATPase, which could influence stomatal movements.

### Incubation Chamber Design and Microenvironment

The prepared leaf materials were mounted into a gas-tight cuvette (Type 7937, ADC Bioscientific, UK) attached to a microscope (Leica, Leitz, DMRX, Wetzlar, Germany) (Supplementary Figure 1). The cuvette was constructed from two aluminium blocks, similar to that described in the study by Shope et al. (2008), giving a total sample volume of 6 cm^3^. The upper section of the cuvette was connected to the microscope lens with a condom, which allowed stomatal images to be recorded whilst maintaining the gaseous environment. The lower section of the cuvette contained a 3.5 cm diameter optical window to allow sample illumination. The cuvette was unstirred, using an internal plenum around the diameter of the cuvette to deliver mixed gas flow. The temperature of the cuvette was maintained at 23 ± 1°C by a chilled water bath supplying integral water jackets in both the upper and lower cuvette sections. The concentration of CO_2_ inside the cuvette was controlled using an infrared gas analyser (IRGA 6400, Licor, NE, USA) at a flow rate of 500 μmol s^−1^ and vapour pressure deficit of 1 ± 0.1 KPa.

The segment of intact leaf and epidermal-mesophyll transfer material was illuminated through the optical window of the lower cuvette section. A white light source (XBO 75 W/HBO 100 W, Leica, Wetzlar, Germany) delivered a light intensity of 400 ± 10 μmol m^−2^ s^−1^ PAR at the surface of the epidermal peel. However, to allow measurements of stomatal opening during dark experimental periods, this was switched to a green LED light source (Luxeon Star, Lumileds Holding B.V., CA, USA) at an actinic intensity of 100 ± 10 μmol m^−2^ s^−1^ PAR which resulted in an intensity of 10 μmol m^−2^ s^−1^ on the abaxial surface (as shown in Supplementary Figure 2). Greenlight was selected in order to minimise photosynthesis and the associated changes in [CO_2_], and although several studies have suggested that stomata can respond to green light, these are mostly associated with reversal of blue light responses (Talbott et al., 2002) or minimal opening responses compared with other wavelengths (Wang et al., 2011). One stoma was measured per day, and all response curves were started between 8:30 and 8:40 a.m. and finished between 2:45 and 3:00 p.m., respectively.

In all of the experiments, the light was changed sequentially from 400 to 0 μmol m^−2^ s^−1^ for 60 min and then returned back to 400 μmol m^−2^ s^−1^, at a stable [CO_2_] of 120 μmol mol^−1^, after which [CO_2_] was changed sequentially from 120 to 650 μmol mol^−1^ for 60 and then back to 120 μmol mol^−1^. Afterward, 60 min was allowed for stomata to respond to darkness or higher [CO_2_]. A low initial [CO_2_] was used to ensure that stomata in all tissues experienced a similar [CO_2_], as [CO_2_] in intact leaves or grafted material would have reduced internal CO_2_ due to photosynthetic CO_2_ drawdown.

### Determination of Stomatal Aperture

Stomatal apertures were measured using a camera (Bresser-Mikrocam 5-megapixel camera, 2,592 × 1,944, Rhede, Germany) attached to the microscope and connected to a personal computer (Supplementary Figure 2). Digital imaging software (Image J; U.S. National Institutes of Health, MD, USA, https://imagej.nih.gov/ij/) was used to measure apertures following calibration with a stage graticule. Each figure shows the results from three to four experiments conducted on different plants of the same age maintained under identical growth environments. As only a single stoma could be measured in the field of view, measurements were conducted over multiple days (3–4) and the data was used to generate mean responses.

### Statistical Analyses

The data are shown as the means ± SE of three or four independent experiments. Possible differences among the mean values of data were analysed using ANOVA-factorial, and the means were compared with a Newman-Keuls test. The data were analysed using Statistica 8 (StatSoft. Inc., Tulsa, OK 74104, USA).

## Results

### Titratable Acidity

In Crassulacean acid metabolism species, titratable acidity (TA) correlated directly with nocturnal CO_2_ fixation and associated malic acid accumulation, and light period malate decarboxylation (Borland et al., 2009). Therefore, we measured titratable acid (TA) at dawn and dusk in order to determine the degree of CAM. As a direct confirmation that the *K. fedtschenkoi* leaves were operating the CAM pathway, the TA content was highest at dawn in the CAM leaves, approximately double the content of CAM leaves at dusk (Supplementary Figure 3). By contrast, only a negligible quantity of TA was measured in C_3_ *V. faba* leaves, and the level did not vary markedly between dawn and dusk (Supplementary Figure 3). No significant differences were observed in the temporal variations in TA for the plants grown in either of the two chambers (“light” and “dark”), demonstrating that the key metabolic correlates of CAM in the *K. fedtschenkoi* leaf mesophyll were likely to be identical regardless of whether the plants were experiencing their dark period between 8:00 p.m. and 08:00 a.m. (“light” chamber), or between 8:00 a.m. and 8:00 p.m. (“dark” chamber).

### C_3_ and CAM Stomatal Responses of Plants in the “Light” Growth Chamber

**Figure 1:**
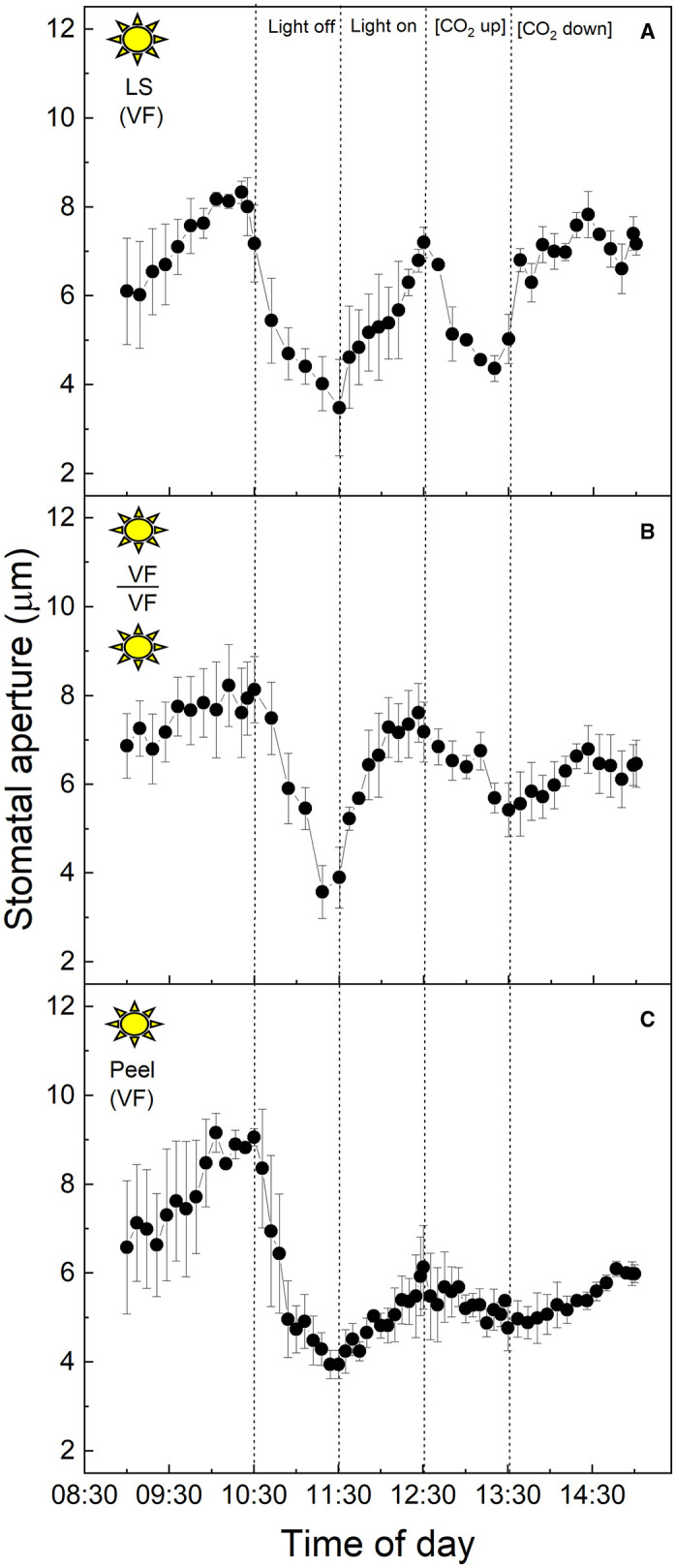
**(A)** *Vicia faba* leaf segment; **(B)** Abaxial peeled epidermis of *Vicia faba* leaf on abaxial exposed mesophyll of *Vicia faba*, from the “light” growth chamber; **(C)** Abaxial isolated epidermis of *Vicia faba* leaf, from the “light” growth chamber (normal light period 8:00 a.m. to 8:00 p.m.). Light intensity was changed from photon flux density of 400 ± 10 μmol m^−2^ s^−1^ to darkness, as indicated, and [CO_2_] was changed from 120 to 650 μmol mol^−1^, as indicated. The temperature of the chamber was maintained at 23 ± 1°C. The values are means of four repetitions (± SE). The sun symbol represents plants taken from the light-grown chamber.

To examine the C_3_ physiology of *V. faba*, peeled epidermises transplanted back onto exposed *V. faba* mesophyll, stomatal responses in both intact leaf segments (Figure 1A), and abaxial epidermal peels placed onto the exposed mesophyll of a different leaf (Figure 1B) were assessed. In addition, epidermal peels in which the mesophyll was completely removed were also measured (Figure 1C). Stomata of *V. faba* sampled from the “light” chamber responded to light intensity as expected for a C_3_ species (Figure 1). Stomatal aperture increased during the first incubation phase with 400 μmol m^−2^ s^−1^ light intensity, with apertures reaching about 8 μm, followed by a decrease in aperture to *ca*. 4 μm when the light was turned off (Figure 1). Restoration of the light to 400 μmol m^−2^ s^−1^ resulted in apertures returning to values close to those before the dark treatment. Exposure to 1 h high CO_2_ (650 μmol mol^−1^) decreased aperture by around 42%, returning to initial values when [CO_2_] was returned to the original level of 120 μmol mol^−1^ (Figure 1). The magnitude of the changes in the stomatal aperture in response to changing light intensity was similar in both the intact leaf segments and the epidermal-mesophyll peeled and transplanted material, illustrating that removing the epidermis from *V. faba* and transplanting it onto exposed mesophyll of an equivalent leaf had little effect on the ability of stomata to function or any influence on a potential putative mesophyll signal (Figure 1B). Isolated epidermis responded to both changing light intensity and [CO_2_] concentration, similar to the intact leaf segments (Figure 1C), although the responses in the latter part of the experiment were dampened.

**Figure 2:**
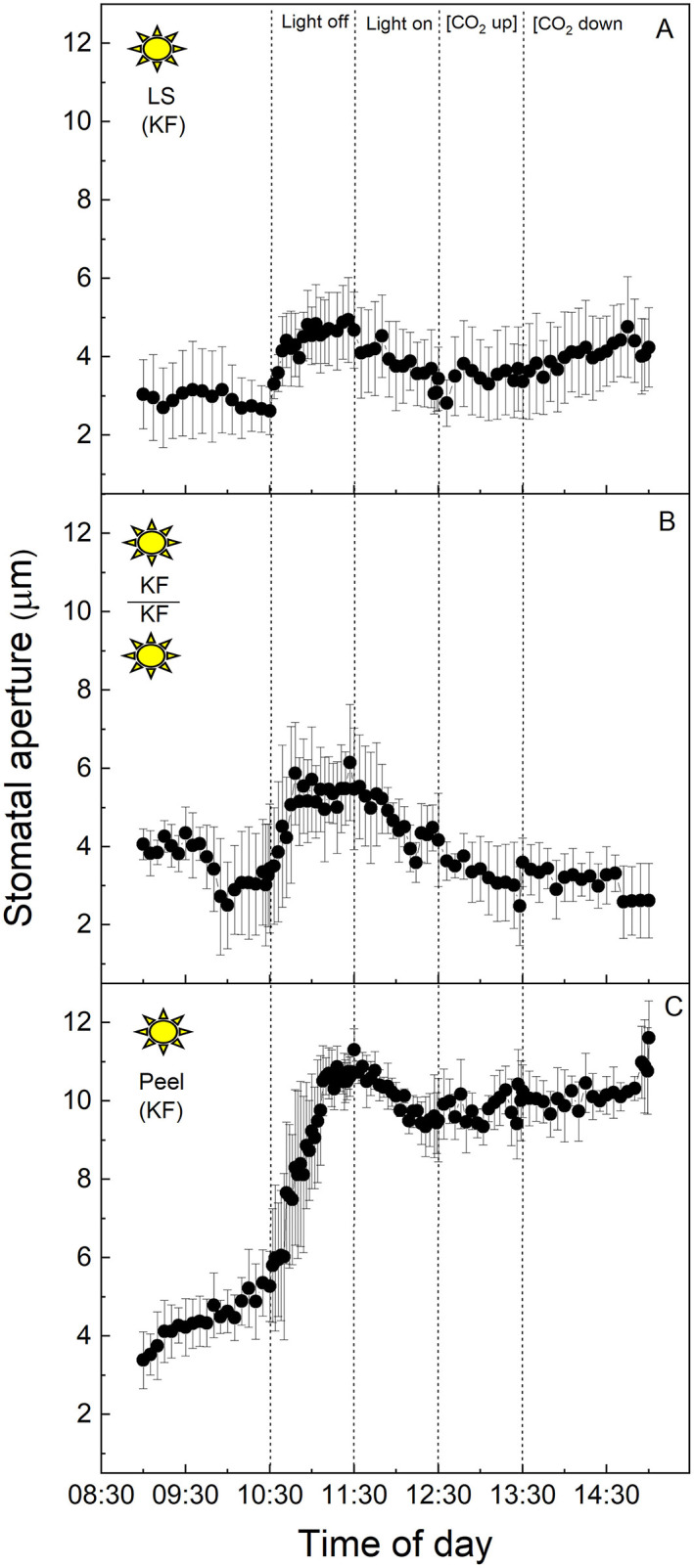
**(A)** *Kalanchoë fedschenkoi* leaf segment; **(B)** Abaxial peeled epidermis of *Kalanchoë fedschenkoi* leaf on abaxial exposed mesophyll of *Kalanchoë fedschenkoi*, from the “light” growth chamber; **(C)** Abaxial isolated epidermis of *Kalanchoë fedschenkoi* leaf, from “light” growth chamber (light period 8:00 a.m. to 8:00 p.m.). Light intensity was changed from photon flux density of 400 ± 10 μmol m^−2^ s^−1^ to darkness, as indicated, and [CO_2_] was changed from 120 to 650 μmol mol^−1^, as indicated. The temperature of the chamber was maintained at 23 ± 1°C. The values are means of four repetitions (± SE). The sun symbol represents plants taken from the light-grown chamber.

The equivalent experiment to that described above was performed using full CAM leaves of *K. fedtschenkoi* sampled from the “light” chamber (Figure 2). In this species, stomatal apertures increased by ~40% upon dark treatment of the intact leaf segment (Figure 2A), or when the abaxial epidermis from one leaf was transplanted onto the mesophyll of a different, but equivalent leaf, and switched into darkness (Figure 2B). The aperture returned to the initial lower value of about 4 μm when the light was turned on again (Figure 2B). Increasing [CO_2_] from 120 to 650 μmol mol^−1^ resulted in a slight decrease in the aperture in the epidermal-mesophyll transfer material in the light (Figure 2B), but the little stomatal movement was observed in the intact leaf segment (Figure 2A). As discussed above, stomata within leaf epidermal peels placed on exposed mesophyll showed similar responses to the intact leaf segment, supporting the conclusion that peeling the epidermis and transplanting it onto exposed mesophyll resulted in the expected stomatal responses for a CAM species, which were observed for the intact leaf segments (*cf .* Figures 2A,B). Interestingly, under these experimental conditions, stomata of the CAM species appeared to be less sensitive to changing [CO_2_] as compared with their response to light intensity, and when compared with stomatal responses to [CO_2_] in C_3_ *V. faba* (Figure 1). Isolated epidermises again showed similar responses to both changing light intensity and [CO_2_] concentration as an intact leaf segment, although the initial dark and low [CO_2_] induced opening was dramatically more pronounced (Figure 2C).

**Figure 3:**
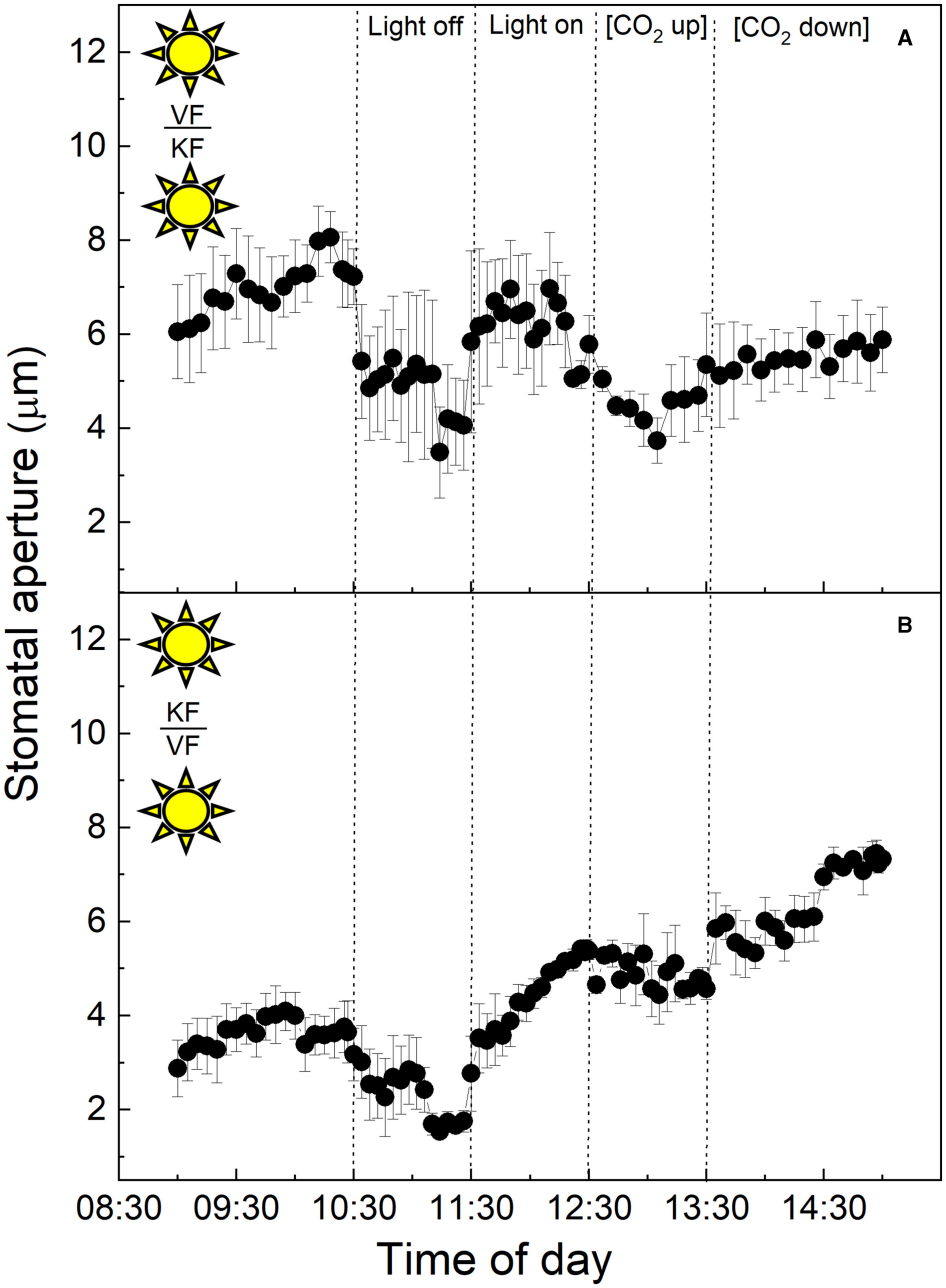
**(A)** Abaxial peeled epidermis of *Vicia faba* leaf on abaxial exposed mesophyll of *Kalanchoë fedschenkoi* leaf from the “light” growth chamber; **(B)** Abaxial peeled epidermis of *Kalanchoë fedschenkoi* leaf on abaxial exposed mesophyll of *Vicia faba* leaf, from the “light” growth chamber (light period 8:00 a.m. to 8:00 p.m.). Light intensity was changed from photon flux density of 400 ± 10 μmol m^−2^ s^−1^ to darkness, as indicated, and [CO_2_] was changed from 120 to 650 μmol mol^−1^, as indicated. The temperature of the chamber was maintained at 23 ± 1°C. The values are means of four repetitions (± SE). The sun symbol represents plants taken from the light-grown chamber.

To investigate the influence of CAM mesophyll tissue on epidermal peels from the C_3_ species *V. faba*, isolated epidermises of the C_3_ species were grafted onto the exposed mesophyll of CAM *K. fedtschenkoi* leaves, and *vice versa* (Figure 3). When C_3_ *V. faba* epidermises were transplanted onto *K. fedtschenkoi* mesophyll, the *V. faba* stomata proceeded to close in response to “light off” and opened when the light was switched on again (Figure 3A). This was consistent with the result observed when *V. faba* epidermis was transplanted onto the exposed *V. faba* mesophyll of an equivalent leaf (Figure 1B). Stomata in epidermal peels from CAM leaves of *K. fedtschenkoi* exhibited a different behaviour when transplanted onto exposed C_3_ mesophyll from *V. faba* (Figure 3B). Under these conditions, the *K. fedtschenkoi* CAM stomata closed in response to darkness, which was the opposite of the behaviour of CAM intact leaves, peels, or when CAM peels were placed on CAM mesophyll (Figure 2). When light intensity was increased, a strong increase in the aperture was observed, which was reduced under high [CO_2_] (albeit less than in the C_3_ responses in Figure 1), and, once again, aperture increased when [CO_2_] was lowered (Figure 3B).

### C_3_ and CAM Stomatal Apertures in the Reverse Growth Chamber

**Figure 4:**
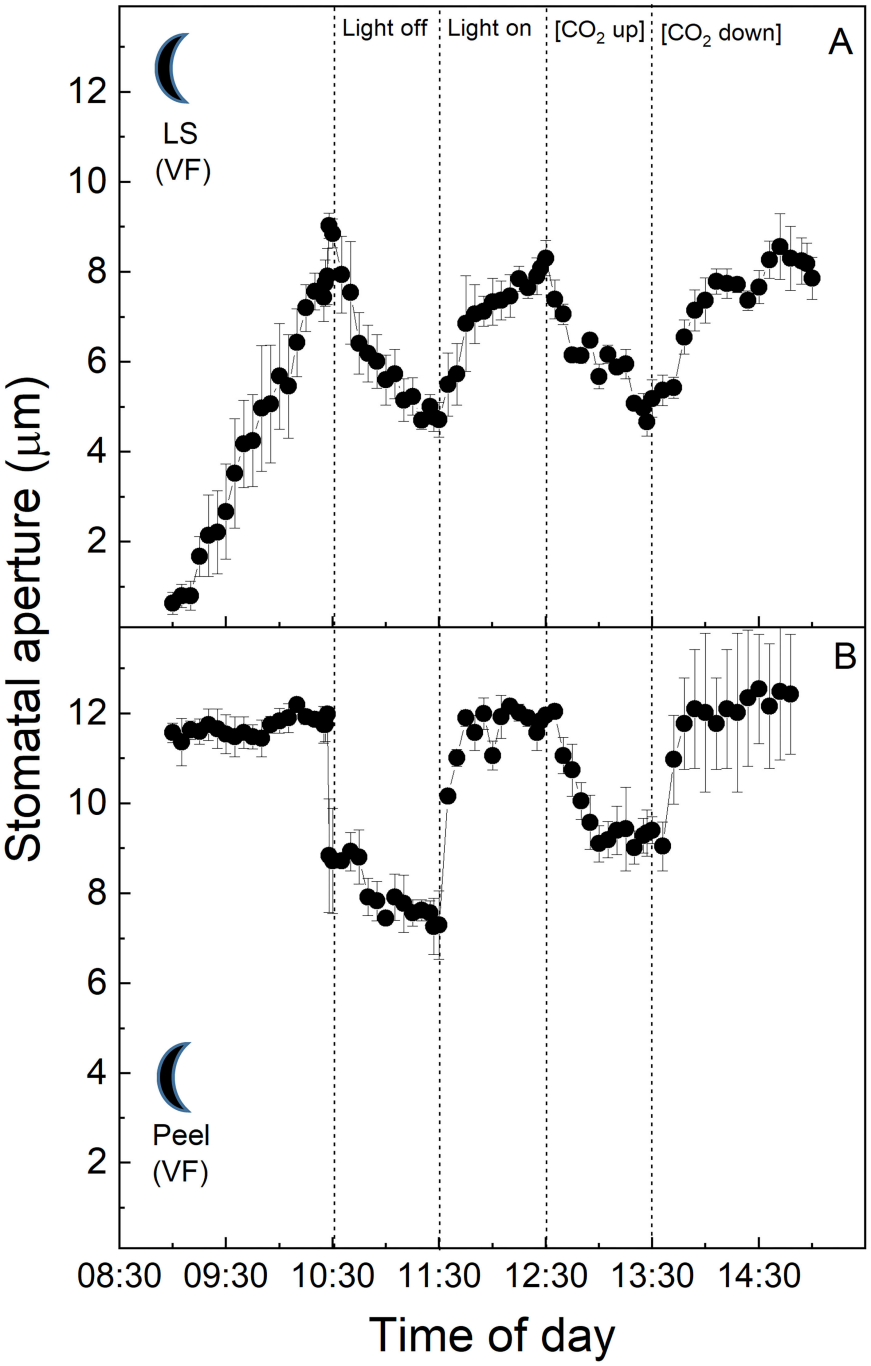
**(A)** *Vicia faba* leaf fragment from the “dark” cabinet.; **(B)** Abaxial isolated epidermis of *Vicia faba* leaf from the “dark” growth chamber (reverse light period 8:00 p.m. to 8:00 a.m.). Light intensity was changed from photon flux density of 400 ± 10 μmol m^−2^ s^−1^ to darkness, as indicated, and [CO_2_] was changed from 120 to 650 μmol mol^−1^, as indicated. The temperature of the chamber was maintained at 23 ± 1°C. The values are means of four repetitions (± SE). The moon symbol represents plants taken from the dark-grown chamber.

**Figure 5:**
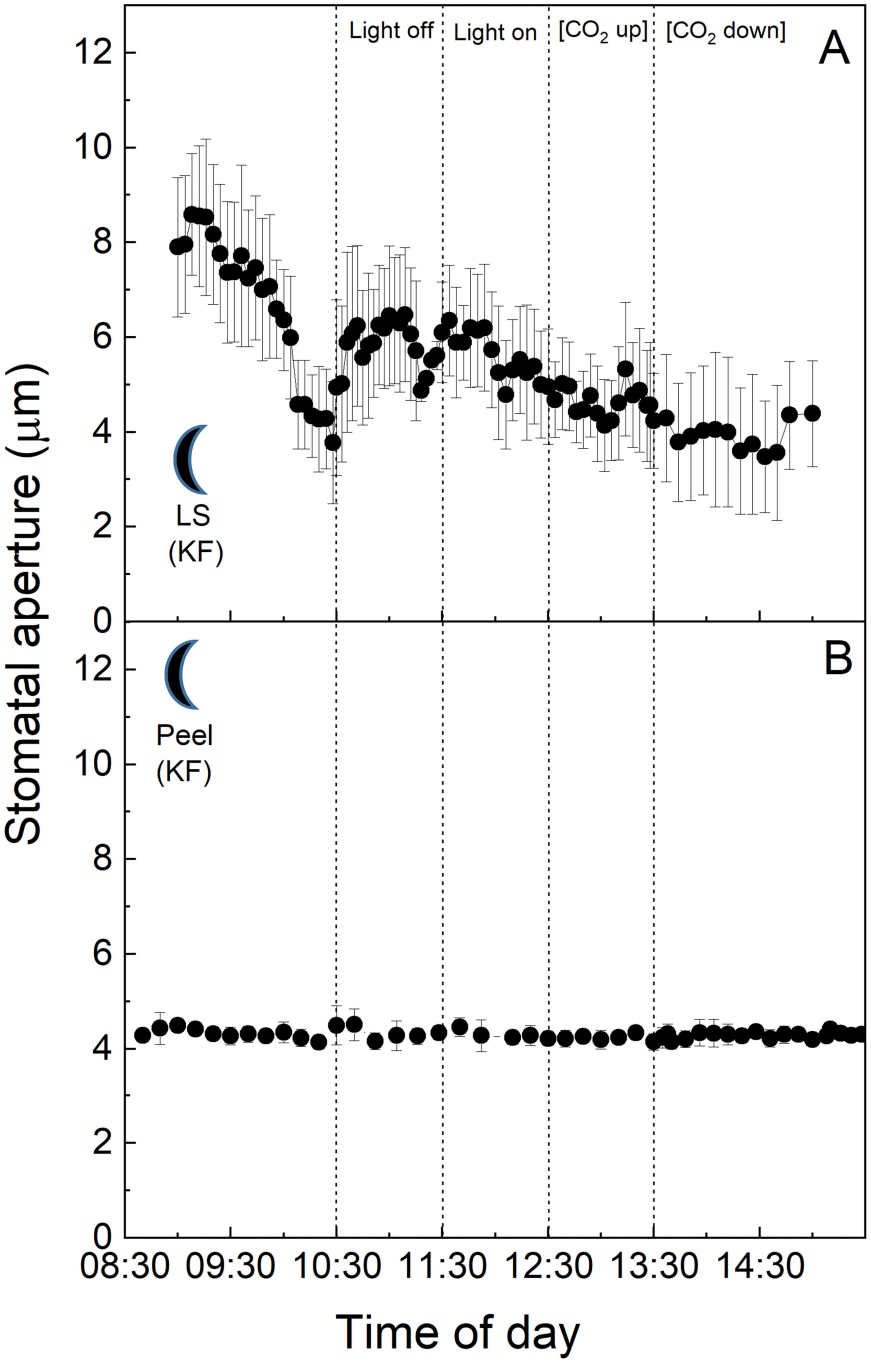
**(A)** *Kalanchoë fedschenkoi* leaf segment from the “dark” growth chamber; **(B)** Abaxial isolated epidermis of *Kalanchoë fedschenkoi* leaf, from the “dark” growth chamber (light period 8:00 p.m. to 8:00 a.m.). Light intensity was changed from photon flux density of 400 ± 10 μmol m^−2^ s^−1^ to darkness, as indicated, and [CO_2_] was changed from 120 to 650 μmol mol^−1^, as indicated. The temperature of the chamber was maintained at 23 ± 1°C. The values are means of four repetitions (± SE). The moon symbol represents plants taken from the dark-grown chamber.

**Figure 6:**
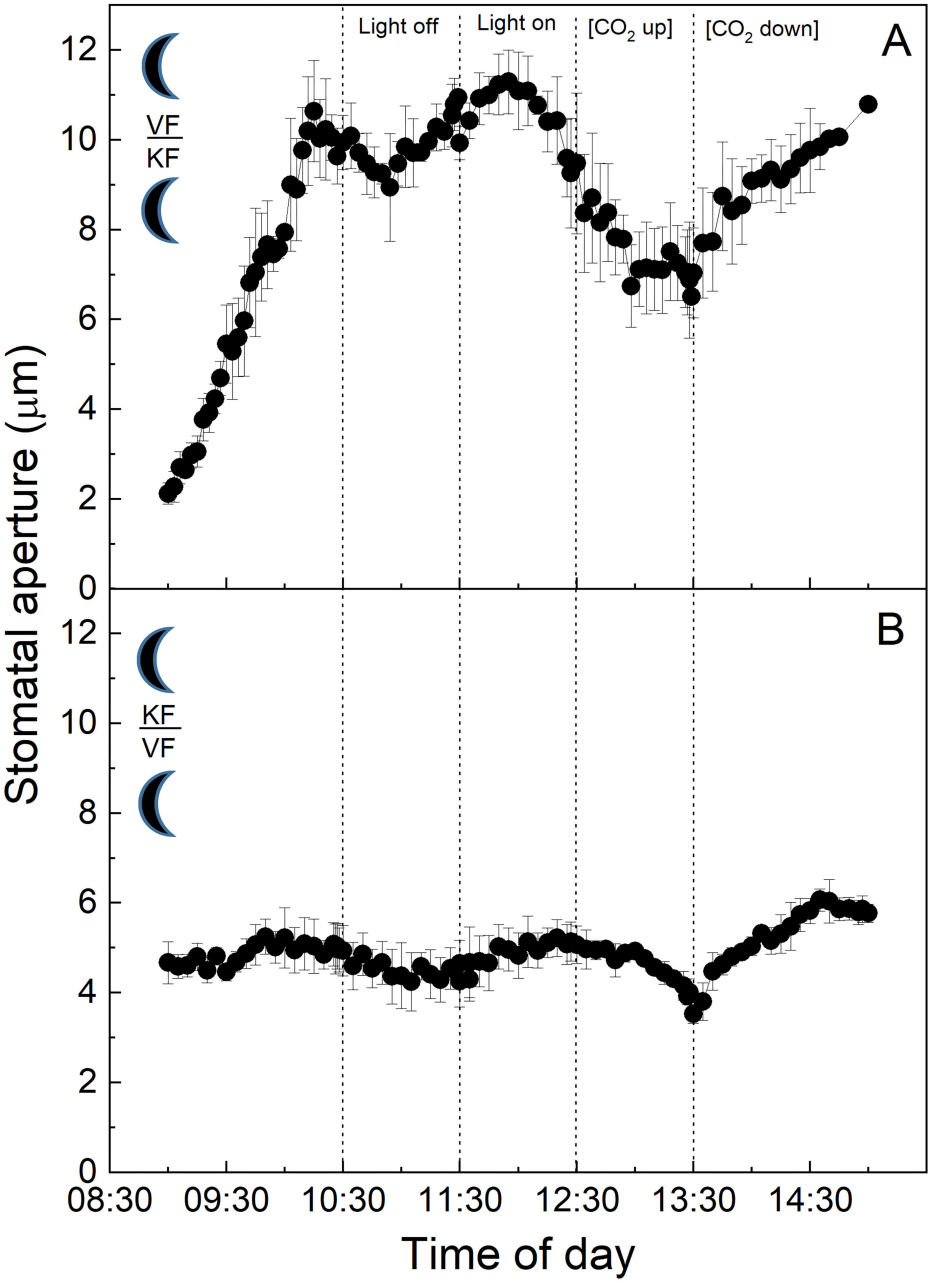
**(A)** Abaxial peeled epidermis of *Vicia faba* leaf on abaxial exposed mesophyll of *Kalanchoë fedschenkoi* leaf both from the “dark” growth chamber; **(B)** Abaxial peeled epidermis of *Kalanchoë fedschenkoi* leaf on abaxial exposed mesophyll of *Vicia faba* leaf from the “dark” growth chamber (reverse light period 8:00 p.m. to 8:00 a.m.). Light intensity was changed from photon flux density of 400 μmol m^−2^ s^−1^ to darkness, as indicated, and [CO_2_] was changed from 120 to 650 μmol mol^−1^, as indicated. The temperature of the chamber was maintained at 23°C. The values are means of four repetitions (± SE). The moon symbol represents plants taken from the dark-grown chamber.

All of the experiments outlined above were carried out using leaves from plants grown in the “light” chamber, lights came on at 8:00 a.m. and switched off at 8:00 p.m. As described above, CAM physiology is very different from that of C_3_ plants, with stomata opening for atmospheric CO_2_ uptake and primary fixation during the nocturnal phase of the diel cycle. Due to this, the next set of experiments were performed on plants entrained in a reverse phase “dark” growth chamber in which the 12-h dark period (8:00 a.m. to 8:00 p.m.) corresponded with the 12-h light period in the “light” chamber, and vice versa. The light period was from 8:00 p.m. to 8:00 a.m. in the “dark” chamber, when the normal chamber had 12-h dark (Figures 4–**6**).

*Vicia faba* C_3_ stomata in the intact leaf segments showed the same behaviour as in the first experiment using the plants from the “light” chamber, although here aperture values were near zero at the start of the experiments, which was consistent with the dark conditions inside the growth chamber prior to the sampling of the leaves for the experiments (cf. Figure 4A with Figure 1A). Furthermore, the *V. faba* epidermal peel without mesophyll showed a similar response to the intact leaf fragment, with the exception of the start of the experiment, when the peels showed a high and stable conductance compared with the leaf segment, which initially had much lower apertures and increased with time in the light (Figure 4A). It was notable that the responses to light in the dark period samples were faster and of a greater magnitude (Figure 4B) when compared with the epidermal peels from plants entrained in the “light” chamber (Figure 1C).

At the start of the experiment, *K. fedtschenkoi* CAM stomata in the intact leaf segments from the “dark” cabinet showed some of the highest initial stomatal aperture values observed among the treatments (*ca*. 8 μm, Figure 5A). Stomata responded to the higher light intensity of the cuvette by closing, then opened slightly when the light was turned off, and subsequently closed during the latter part of the experiment when [CO_2_] was altered. Surprisingly, no response to either light intensity or [CO_2_] was observed in the dark sampled CAM epidermal peels from the “dark” chamber (Figure 5B).

When the epidermis was removed at the start of the dark period from plants grown in the “dark” growth conditions and placed on the mesophyll of plants grown in the “dark” conditions, stomata of the C_3_ *V. faba* epidermis were placed onto *K. fedtschenkoi* exposed mesophyll performing CAM showed the same behaviour as stomata in C_3_ intact leaves in the dark (Figures 4A, 6A). In contrast, stomata in the CAM epidermis exposed to C_3_ mesophyll from the “dark” chamber showed limited response to changes in light intensity (Figure 6B), unlike plants grown in the “light” cabinets (Figure 3B). A dampened, but typical C_3_ CO_2_ response was observed, with aperture decreasing slightly with increasing [CO_2_] and opening when [CO_2_] was lowered (Figure 6B), which was similar to the CO_2_ response observed in the light-grown material (Figure 3B).

**Figure 7:**
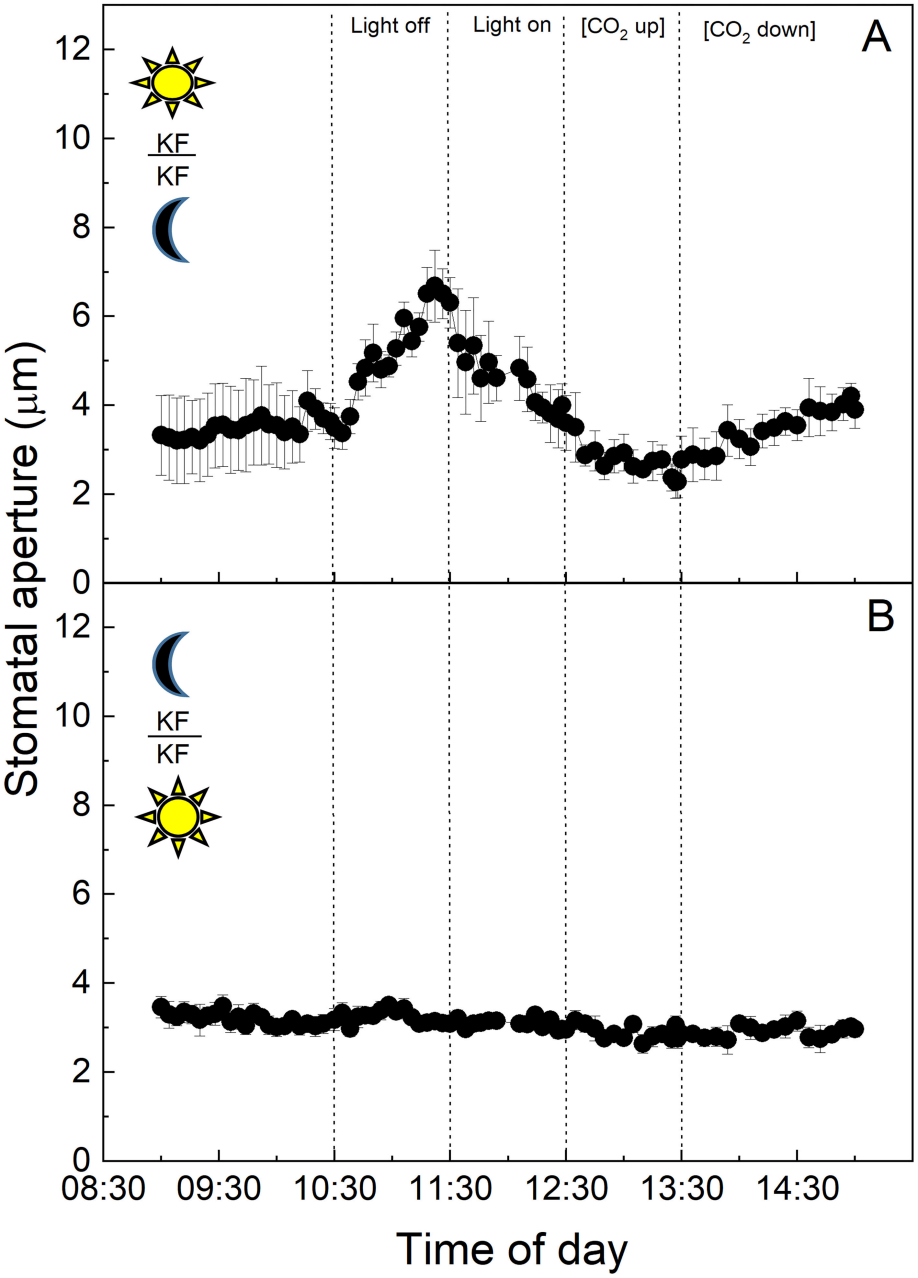
**(A)** Abaxial peeled epidermis of *Kalanchoë fedschenkoi* leaf from the “light” growth chamber, on abaxial exposed mesophyll of *Kalanchoë fedschenkoi* leaf, from the “dark” growth chamber; **(B)** Abaxial peeled epidermis of *Kalanchoë fedschenkoi* leaf from the “dark” growth chamber, on abaxial exposed mesophyll of *Kalanchoë fedschenkoi* leaf from the “light” growth chamber (light period 8:00 a.m. to 8:00 p.m.), and from the “dark” growth chamber (reverse light period 8:00 p.m. to 8:00 a.m.). Light intensity was changed from photon flux density of 400 ± 1 0 μmol m^−2^ s^−1^ to darkness, as indicated, and [CO_2_] was changed from 120 to 650 μmol mol^−1^, as indicated. The temperature of the chamber was maintained at 23 ± 1°C. The values are means of four repetitions (± SE). The sun symbol represents plants taken from the light-grown chamber and the moon symbol represents plants taken from the dark-grown chamber.

Finally, when the epidermis from CAM *K. fedtschenkoi* leaves grown in the “light” cabinet was placed on mesophyll from the “dark” cabinet and subjected to changes in light intensity and [CO_2_], stomata showed the typical CAM response to light by increasing aperture in darkness and decreasing aperture when light intensity was increased (Figure 7A). In response to high [CO_2_], stomata closed, although this response was not of the same magnitude as the response to light (Figure 7A). However, when the epidermis from dark sampled CAM leaves grown in the “dark” cabinet was placed on mesophyll from the “light” cabinet and subjected to the same light and CO_2_ changes, stomata showed no response to light or changing [CO_2_], with stomatal aperture maintained <4 μm (Figure 7B).

## Discussion

In this study, we compared stomatal responses and the influence of mesophyll on stomatal aperture in two species with different types of photosynthetic metabolism, namely *V. faba* (C_3_) and *K. fedtschenkoi* (CAM). We showed that stomata from isolated peels (in which the influence of mesophyll had been removed) behaved in a similar manner (although sometimes with different magnitudes for response) to both intact leaf segments and epidermal transfers, with exception of those from the CAM “dark” chamber (Figure 5B). Stomatal responses observed in the C_3_ *V. faba* samples were as expected (Olsen et al., 2002; Fujita et al., 2013), with a characteristic opening in response to increasing light intensity, closure in response to dark, and closure at high [CO_2_] (Lawson and Blatt, 2014). In contrast, the stomatal aperture in the CAM leaf sections/peels sampled in the light, as well as intact leaf segments sampled in the dark, increased in response to darkness (Figures 2, 5A), although it was noteworthy that dark sampled *K. fedtschenkoi* epidermal peels did not respond to changes in light or [CO_2_] (Figure 5B). To date, we are unaware of any previous studies that reported stomatal opening as a direct response to darkness in CAM plants, and the fact that this could be observed in isolated peels from CAM leaves sampled in the light (Figure 2C) suggested that light intensity is perceived by the guard cells themselves. Although it was well established that CAM plants open stomata in the dark period (Cockburn, 1983), this has been associated with the reduction in *C*_*i*_ when PPC activity in the mesophyll increases at dusk (Wyka et al., 2005; Griffiths et al., 2007; von Caemmerer and Griffiths, 2009). Similarly, stomatal closure during the light period was thought to be driven by the generation of internal CO_2_ (increased *C*_*i*_) due to the decarboxylation of stored malic acid (Cockburn et al., 1979; Spalding et al., 1979). The above theoretical framework for understanding the physiological responses of stomata in CAM species implied that a mesophyll signal (including *C*_*i*_) was required for stomatal responses to changes in light and [CO_2_], which was not fully supported by our findings.

Specifically, the findings presented here suggest the presence of additional autonomous guard cell behaviour in the guard cell pairs of CAM leaves of *K. fedtschenkoi*. However, this can be overridden by the presence of C_3_ mesophyll (Figure 3B), indicating that stomatal behaviour in CAM plants can be influenced by a signal transmitted from the mesophyll in stomatal responses to light intensity, as has been proposed for C_3_ and C_4_ plants (Shimazaki et al., 2007).

### Stomatal Responses to Changes in Light Intensity

In C_3_ and C_4_ plants, stomatal responses to light are divided into two categories (Matthews et al., 2020). The first is the red light or photosynthetic response, which is dependent on mesophyll and/or guard cell chloroplasts (Mott et al., 2008; Suetsugu et al., 2014), and is often closely associated with stomatal responses to *C*_*i*_ as described above (although other signals have been suggested; see Lawson et al., 2014, 2018). The second is the blue light (BL) response. This is independent of photosynthesis and the result of a signalling cascade that starts with the perception of low fluence rates of BL in the guard cells by phototropin (Kinoshita et al., 2001, 2003; Inoue et al., 2008) which triggers the action of the plasmalemma H^+^-ATPase pumps, resulting in hyperpolarization of the plasma membrane and stomatal opening. Interestingly a recent study has shown that guard cell H^+^ -ATPase pumps were also activated by red light and their action correlates with the stomatal opening. However, DCMU abolished this response indicating that a photosynthetic factor in the mesophyll was also required (Ando and Kinoshita, 2018). A subsequent study confirmed red light-driven stomatal opening in epidermal peels of *Commelina communis*, although mesophyll involvement was not essential. However, the presence of mesophyll tissue accelerated stomatal opening (Fujita et al., 2019). The stomatal BL response is species-specific (Vialet-Chabrand et al., 2021), and has been reported to be absent in the facultative CAM species *Mesembryanthemum crystallinum* when the plant shifts from C_3_ metabolism to CAM (Tallman et al., 1997). In contrast to this, a recent study reported BL-dependent stomatal opening in the obligate CAM plants *K. pinnata* and *K. daigremontiana*, and that BL-induced opening was not linked to CO_2_ assimilation (Gotoh et al., 2019). Since our study was conducted using white light on peels, our findings could support CAM guard cell perception of blue (or red) light in *K. fedtschenkoi* leading to a change in aperture (in a different direction to C_3_ and depending on whether the CAM leaf was sampled from the light or dark period). Even more intriguing was the fact that *V. faba* C_3_ mesophyll overrode the stomatal response of the *K. fedtschenkoi* CAM epidermis sampled in the light. This finding supported the proposal that a mesophyll-derived signal can dominate over a guard cell signal resulting in stomata in CAM peels behaving as C_3_ in response to light (Figure 3B). Interestingly, CAM mesophyll was unable to override the C_3_ stomatal response in peels from *V. faba* (Figure 3A).

### The Influence of Mesophyll on Stomatal Responses

Several studies in C_3_ species have shown that a mesophyll signal other than [CO_2_] is required to drive stomatal responses (Mott et al., 2008; Fujita et al., 2013, 2019), and the same may also be true for CAM stomata. Isolated C_3_ epidermal peels responded to changing light intensity and [CO_2_], at a slower and reduced magnitude of response (Figure 1C) supporting the involvement of a mesophyll signal (Mott et al., 2008), but indicated that this is not essential. Other studies have shown that the speed of stomatal responses to light in peels also depends on epidermal turgor pressures (as shown in Zeiger et al., 1987 and references therein). The nature of a mesophyll signal remains unclear, some studies have suggested that guard cell chloroplastic photosynthetic electron transport is involved in C_3_ stomatal behaviour (Olsen et al., 2002; Lawson et al., 2002, 2003; Lawson, 2009), while others suggested a vapour ion (Mott et al., 2014) or aqueous signal (Fujita et al., 2013).

The study of Mott et al. (2008) reported no stomatal responses to light and [CO_2_] in epidermal peels from *Vicia, Tradescantia*, or *Pisum*. However, they also showed that stomatal responses were restored in *Tradescantia* and *Pisum*, but not *Vicia*, when the peels were grafted back onto the underlying mesophyll. The authors used these data to suggest that the mesophyll is responsible for detecting changes in light and [CO_2_] and a mesophyll-driven signal coordinates changes in stomatal aperture. These findings did not entirely agree with our data since we demonstrated a stomatal response in both *Vicia* epidermal peels with and without the mesophyll, although the stomatal response to [CO_2_] was greatly dampened in some peel experiments, suggesting that a mesophyll signal plays a role in the CO_2_ response. A major difference between our experiments and those conducted by Mott et al. (2008) was their use of a 12-h incubation time of epidermal peel samples before use, which may have altered any stored carbohydrates, e.g., starch, within the guard cells, and/or lead to greater stomatal apertures in peels due to hydro-passive effects both of which could have influenced stomatal behaviour. The study of McAdam and Brodribb (2012), using a similar xenografting approach to ours demonstrated that stomata responded to increasing light intensity in isolated peels of angiosperms but closure to decreasing light was not observed and only restored when they were placed back onto their own mesophyll or the mesophyll from ferns. Although our data for the isolated epidermises of *V. faba* illustrated stomata closing in response to decreasing light, the response to increasing light and [CO_2_] after this closure were somewhat dampened. The lower ambient [CO_2_] used in our experiment could explain some of the differences between our experiments and those of McAdam and Brodribb (2012).

In this study, we had shown that CAM stomatal responses to changing [CO_2_] (particularly in material with attached *K. fedtschenkoi* mesophyll) were somewhat dampened compared with the C_3_ response in *V. faba* (Figures 1, 2), and whether the plant was sampled from the dark period or the light period prior to measurements did not influence the responses (Figures 4, 5). The fact that these measurements were performed under illumination and stomata responded to changes in light intensity may suggest that light signals override other signals including those driven by changing [CO_2_] (Lawson et al., 2010). However, when CAM epidermal peels were placed on C_3_ mesophyll (from either light or dark chamber) stomata opened in response to a decrease [CO_2_] (Figures 3B, 6B). The greater sensitivity to [CO_2_] of CAM stomata when grafted onto C_3_ mesophyll could be explained by an enhanced CO_2_ draw-down from C_3_ metabolism, which for *V faba* has photosynthetic rates of around 20 μmol m^−2^ s^−1^ (e.g., Lawson and Blatt, 2014), which was generally greater than *K. fedtschenkoi* which has been reported to be somewhere between 5 and 8 μmol m^−2^ s^−1^ (e.g., Boxall et al., 2020; Ceusters et al., 2021). These findings could also suggest that signals in the CAM mesophyll are *preventing* stomatal opening supporting a role for mesophyll signals as well as *C*_*i*_ in stomatal responses. Several studies support the suggestion, that *C*_*i*_ is not the only and major signal to which CAM stomata respond and that other signals must be involved (von Caemmerer and Griffiths, 2009 Males and Griffiths, 2017).

### Stomatal Aperture Responses Using Leaves Sampled From the Dark seriod

Crassulacean acid metabolism has a completely different diel pattern of gas exchange physiology and associated mesophyll metabolism in comparison with C_3_ plants, plus both the circadian clock, along with malic acid content stored in the mesophyll vacuole, are believed to play important roles in CAM stomatal responses (Wilkins, 1991; Nimmo, 2000; Dodd et al., 2002; Borland et al., 2006; Hartwell, 2006). Due to this distinctive temporal regulation of stomatal physiology associated with CAM, we also repeated the epidermal peel transfer experiments using plants in which the lighting regime had been swapped and grown under a “dark” regime and sampled at the start of the dark period (Figures 4–7). When grown in the “dark” growth chamber, stomata on leaf segments from C_3_ *V. faba* plants showed little difference in their responses compared with the “light” conditions (Figures 1, 4). However, stomatal responses in the isolated epidermis from the “dark” grown plants showed much more rapid responses compared with the whole leaf segment of plants sampled from the “light” regime (Figure 4B). It is noteworthy that maximum stomatal aperture was initially observed in *V. faba* peels from the dark material, which might be due to pre-dawn stomatal opening, or due to biological or technical differences when sampling dark material. Surprisingly stomata in CAM epidermal peels from the “dark” chamber (Figure 5B) were unresponsive to both light and [CO_2_] and this response was not restored by placing the epidermal peels onto CAM mesophyll from the light period (Figure 7B). Stomata in C_3_ *V. faba* peels sampled in the dark period and placed onto dark sampled *K. fedtschenkoi* CAM mesophyll (Figure 6A), showed a typical C_3_ type stomatal response, with relatively large magnitudes of change. The second and third light switch events showed some deviation from the expected results with stomata starting to open even though the light was off, and this is most likely due to lags in stomatal behaviour (Lawson and Matthews, 2020) and/or sluggish stomatal responses in peels that have been reported previously (Lee and Bowling, 1992; Roelfsema et al., 2002). These findings could also indicate a slow CAM mesophyll response. When CAM *K. fedtschenkoi* epidermises sampled from the dark were placed on C_3_ *V. faba* mesophyll from the dark period, the stomatal aperture showed very little response to changing light intensity and remained at a steady aperture (Figure 6B). However, a typical, although small, C_3_ type response was observed with changing [CO_2_]. These data and those from the other experiments presented indicated strongly that stomatal responses to [CO_2_] are influenced by the presence of the mesophyll.

These findings could be explained solely by changes in *C*_*i*_, as light would trigger activation of Calvin cycle enzymes in C_3_ which would drive the mesophyll consumption of CO_2_, reducing *C*_*i*_, which should have elicited changes in stomatal aperture during light-dark transitions (Roelfsema et al., 2002) which were not observed. It is worth noting that, unlike leaves from the “light” growth chamber (Figure 3B), C_3_ mesophyll from plants grown in the “dark” chamber was unable to drive a stomatal response to light (Figure 6B). This provided strong support for the proposal that in the plants from the “dark” chamber, there was a factor missing from either the guard cells themselves, or such a factor needs to be provided from the CAM “dark” mesophyll and is essential for light-driven stomatal behaviour.

To further investigate the influence of the CAM mesophyll/photosynthetic signals on stomatal responses, we conducted a reciprocal epidermal peel transfer experiment in which epidermis from *K. fedtschenkoi* grown in the “light” chamber was placed on CAM mesophyll from plants in the “dark” chamber, and vice versa (Figure 7). In the first part of this experiment, stomata in epidermal peels from the light transplanted onto exposed mesophyll from the dark displaying a typical CAM response (*cf*. Figures 2, 7) to light although the CO_2_ response was dampened. However, when *K. fedtschenkoi* CAM leaf epidermal peels from the dark period were transplanted onto the CAM mesophyll from the “light” cabinet, no stomatal responses to light or [CO_2_] were observed (Figure 7B). Under the first conditions, the *K. fedtschenkoi* mesophyll had just experienced a 12-h dark period and would therefore be transitioning through Phases II and III of CAM, producing internal CO_2_ through decarboxylation of stored malate, which could explain the lack of a stomatal response. However, this was not the case when dark period *V. faba* epidermal peels were placed on dark period CAM mesophyll (Figure 6A), suggesting that C_3_ *V. faba* guard cells in the dark period were not influenced by the *K. fedtschenkoi* CAM mesophyll that they were transplanted onto.

Our findings suggested both a direct mesophyll influence and a guard cell-specific response, depending on the growth environment. The guard cell-specific component was demonstrated for CAM peels sampled from the light period, which responded to light, whereas the lack of any stomatal response in epidermal peels taken from CAM leaves of *K. fedtschenkoi* plants sampled in the dark (Figures 5B, 7B) has supported the requirement for a mesophyll signal, as demonstrated for the intact leaf segment (Figure 5A). Furthermore, the fact *K. fedtschenkoi* peels from the “light” chamber grown plants were able to respond to both light off and the light was switched back on (Figure 2C), suggesting that the light period *K. fedtschenkoi* guard cells had the required stores and/or metabolites and other regulatory/signalling components required for guard cell osmoregulation and that these might be lacking in guard cells sampled from the “dark”'chamber plants (see Lawson et al., 2014, 2018).

Under the conditions of the present study, a logical hypothesis was that stomata would be less responsive, particularly to changes in light intensity, due to the fact that CAM leaves sampled from the “light” growth chamber would possess high levels of malic acid, Furthermore, we hypothesised that leaves sampled from the “dark” growth chamber would open stomata more readily due to the low malic acid content early in the dark period when the experiments were started. However, our findings demonstrated that the mechanism is not as simple as this, as CAM mesophyll sampled from both the light and the dark period stimulated stomatal responses in C_3_ and CAM peels sampled from the light period. Conversely, stomata in *K. fedtschenkoi* epidermal peels from the dark period had no response, supporting the conclusion that both stored products in guard cells and signals from mesophyll cells influence stomatal responses.

In summary, we concluded that guard cells can respond independently of the mesophyll, but this was greatly dampened when underlying mesophyll signals were removed. These results further highlighted the importance of the mesophyll for both the rapidity and the magnitude of the observed stomatal aperture responses. Furthermore, we had demonstrated that mesophyll signals could alter the typical CAM stomatal response, with C_3_ *V. faba* mesophyll tissue able to stimulate the stomata in *K. fedtschenkoi* epidermal peels from CAM leaves sampled in the light growth chamber to behave like those of a C_3_ plant (Figure 3B). Therefore, both mesophyll and guard cell metabolism and/or cell signalling machinery contributed to stomatal responses. Additionally, mesophyll influences were not solely through changes in *C*_*i*_ (although these clearly play an important role) but also through some other unknown signal.

## Data Availability Statement

The raw data supporting the conclusions of this article will be made available by the authors, without undue reservation.

## Author Contributions

MS, AB, JH, and TL discussed and plan the work. MS, PD, and TH conducted the experiments. MS carried out the data analysis, created the figures, and drafted the initial manuscript. MS, TL, JH, and TH wrote the manuscript. All authors commented, made corrections, and approved the submitted version.

## Conflict of Interest

The authors declare that the research was conducted in the absence of any commercial or financial relationships that could be construed as a potential conflict of interest.

## Publisher's Note

All claims expressed in this article are solely those of the authors and do not necessarily represent those of their affiliated organizations, or those of the publisher, the editors and the reviewers. Any product that may be evaluated in this article, or claim that may be made by its manufacturer, is not guaranteed or endorsed by the publisher.

## Supplementary Material

Supplementary Figure 1 Photograph of the microscope system along with leaf section chambers.

Supplementary Figure 2 Examples of images taken using the microscopy system. Image of stoma taken from intact leaf segments from **(A)** *Kalanchoë fedtschenkoi* **(B)** *Vicia faba*, and from epidermal peels of **(C)** *Kalanchoë fedtschenkoi* and **(D)** *Vicia faba*. All scale bars represent 10 mm.

Supplementary Figure 3 Changes in titratable acidity of *Vicia faba* (C_3_) and *Kalanchoë fedschenkoi* (CAM) grown in a “light” growth chamber (light period 8:00 a.m. to 8:00 p.m.) and in reverse time “dark” cabinet (light period 8:00 p.m. to 8:00 a.m.) for local time. All other variables were the same in both chambers: light intensity of 390 μmol m^−2^ s^−1^, the temperature of 18°C (night) and 25°C (day), and air humidity of 60%. The dawn moment was 7:30 a.m. and dusk was 7:30 p.m. The values are means of three repetitions (± SE). Means followed by different letters denote differences among treatments (*P* < 0.005).
