# Supplementary figures and images for: Stomatal Responses to Light, CO2, and Mesophyll Tissue in Vicia faba and Kalanchoë fedtschenkoi
Source: Front Plant Sci. 2021 Oct 27;12:740534. doi: 10.3389/fpls.2021.740534 (PMC8579043; doi:10.3389/fpls.2021.740534)

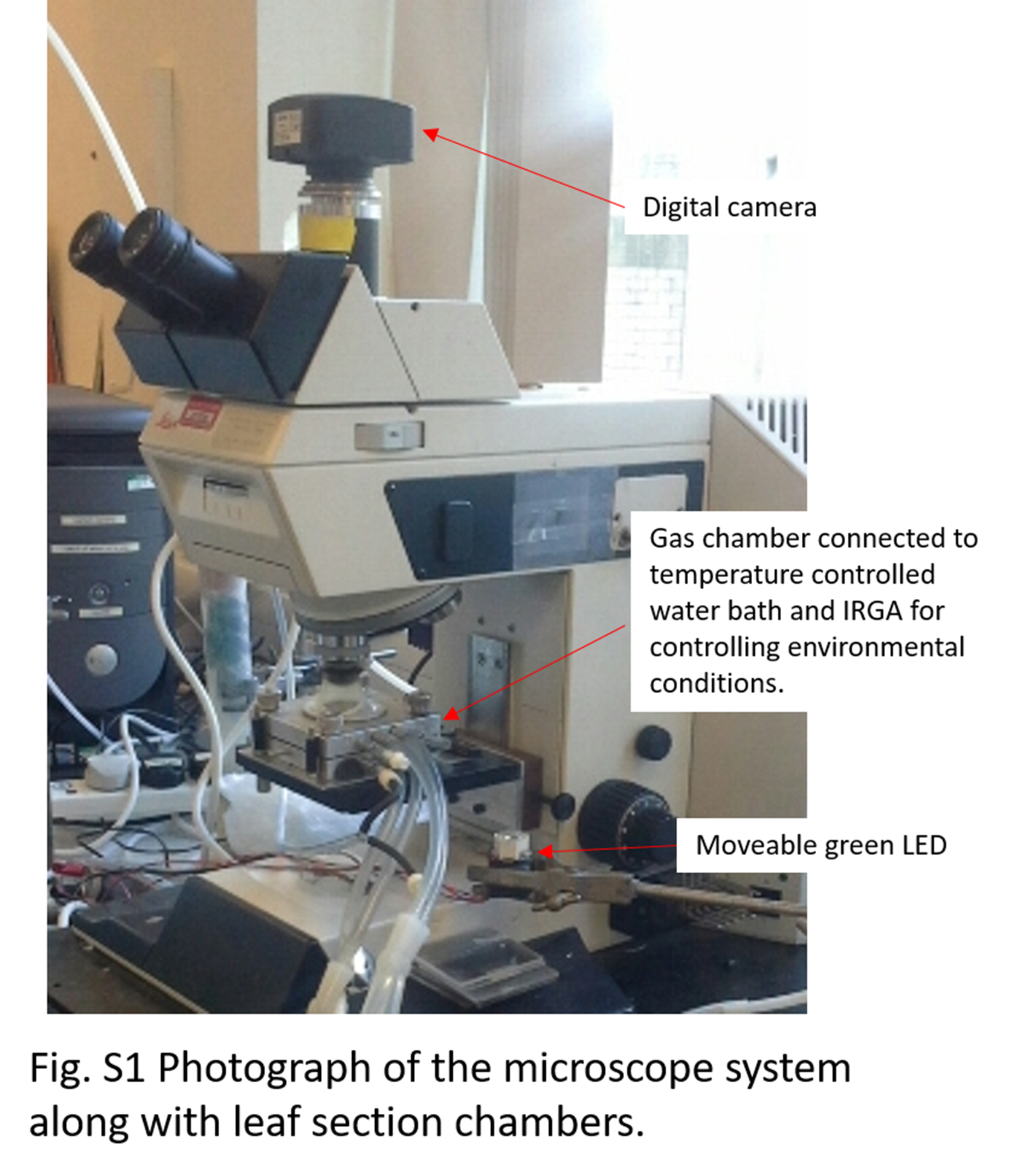

Supplement: Supplementary Figure 1 — Photograph of the microscope system along with leaf section chambers. [file Image_1.TIF]

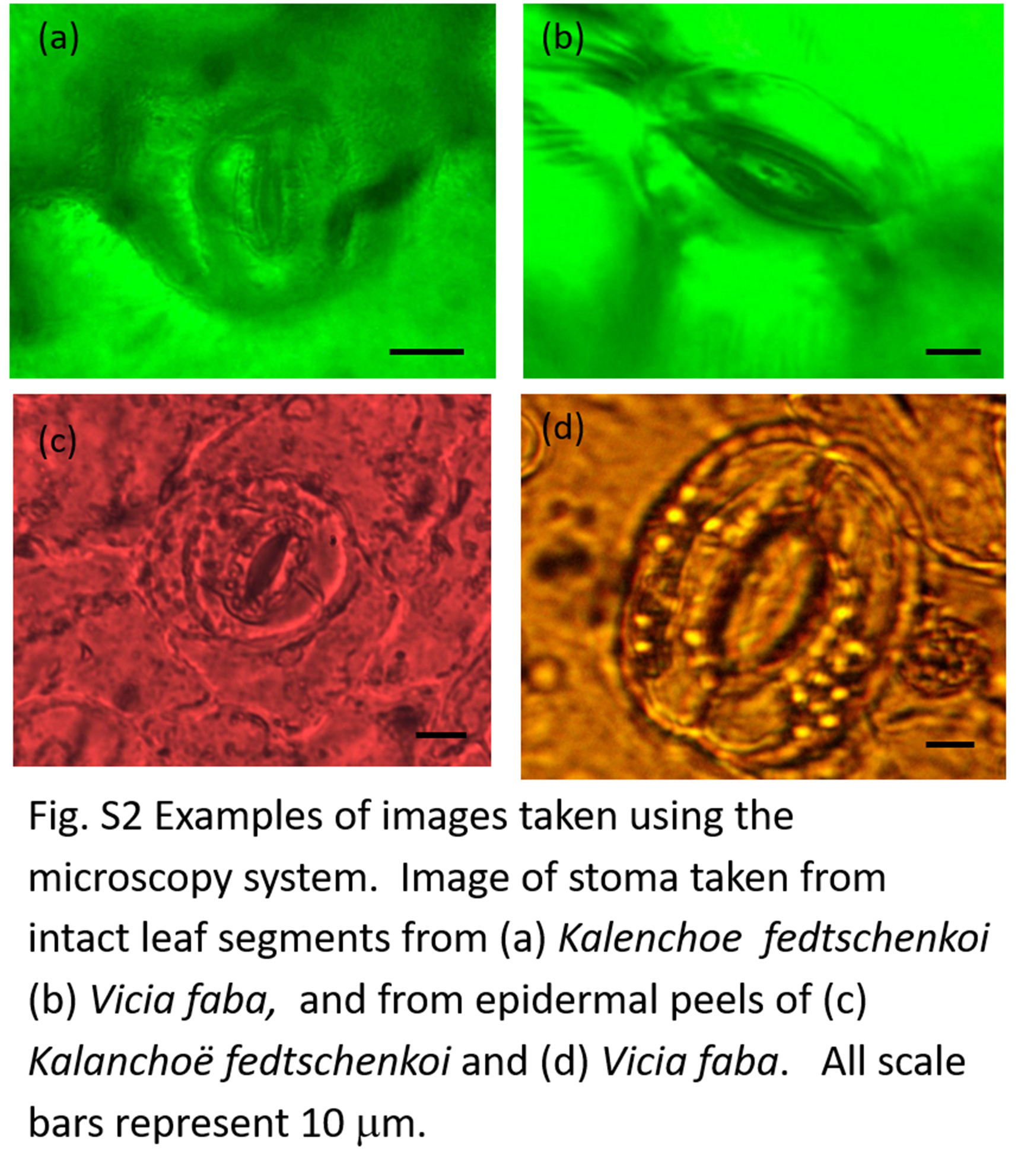

Supplement: Supplementary Figure 2 — Examples of images taken using the microscopy system. Image of stoma taken from intact leaf segments from (A) Kalanchoë fedtschenkoi (B) Vicia faba, and from epidermal peels of (C) Kalanchoë fedtschenkoi and (D) Vicia faba. All scale bars represent 10 mm. [file Image_2.TIF]

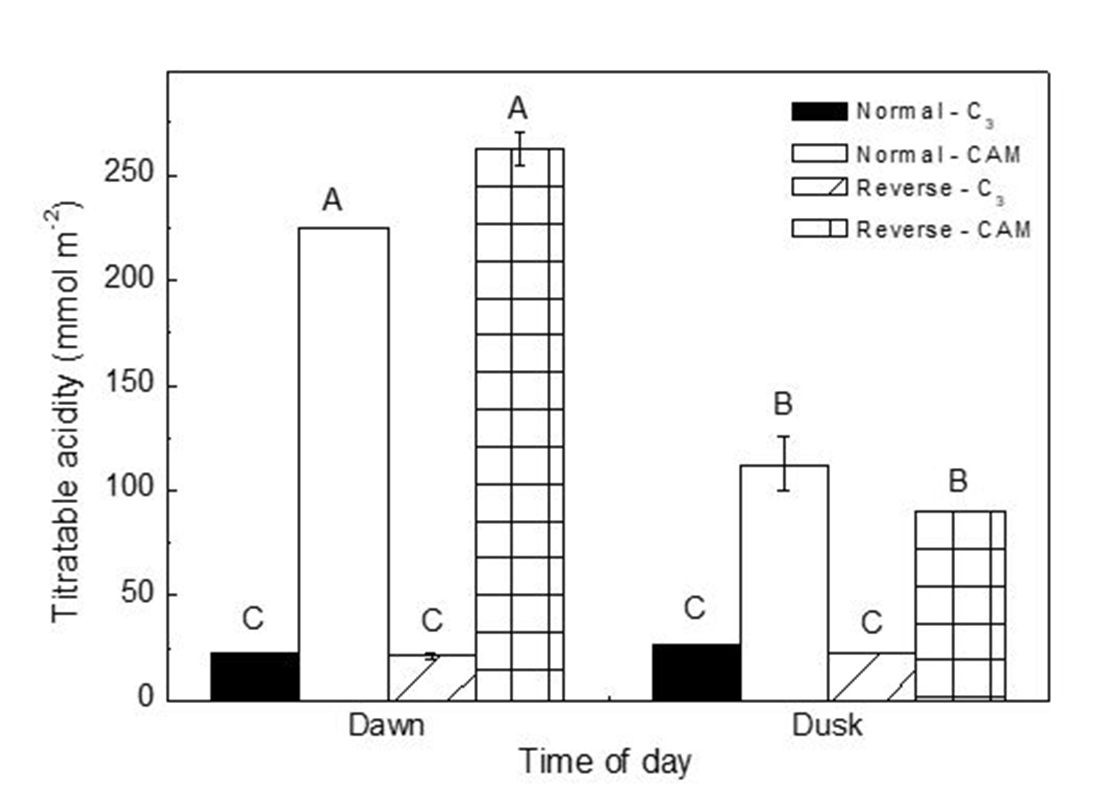

Supplement: Supplementary Figure 3 — Changes in titratable acidity of Vicia faba (C3) and Kalanchoë fedschenkoi (CAM) grown in a “light” growth chamber (light period 8:00 a.m. to 8:00 p.m.) and in reverse time “dark” cabinet (light period 8:00 p.m. to 8:00 a.m.) for local time. All other variables were the same in both chambers: light intensity of 390 μmol m−2 s−1, the temperature of 18°C (night) and 25°C (day), and air humidity of 60%. The dawn moment was 7:30 a.m. and dusk was 7:30 p.m. The values are means of three repetitions (± SE). Means followed by different letters denote differences among treatments (P < 0.005). [file Image_3.JPEG]
